# Tuning out emotional distractors by tuning in theta rhythm: improving visual working memory using transcranial magnetic stimulation

**DOI:** 10.1093/scan/nsag063

**Published:** 2026-08-04

**Authors:** François Thiffault, Benoit Brisson, Isabelle Blanchette

**Affiliations:** Department of Psychology, Université du Québec à Trois-Rivières, Trois-Rivières, QC G9A 5H7, Canada; Laboratoire de Neurosciences Cognitives et Psychologie Inclusive—NeCπ, Université du Québec à Trois-Rivières, Trois-Rivières, QC G9A 5H7, Canada; Department of Psychology, Université du Québec à Trois-Rivières, Trois-Rivières, QC G9A 5H7, Canada; Laboratoire de Neurosciences Cognitives et Psychologie Inclusive—NeCπ, Université du Québec à Trois-Rivières, Trois-Rivières, QC G9A 5H7, Canada; School of Psychology, Faculty of Social Sciences, Université Laval, Québec, QC G1V 0A6, Canada

**Keywords:** emotion, visual working memory, transcranial magnetic stimulation, theta rhythm, event-related potential

## Abstract

The relation between attentional control and visual working memory (vWM) has previously been studied with transcranial magnetic stimulation (TMS). For example, it has been shown that rhythmic TMS (rhTMS) can enhance attentional control and, consequently, vWM performance, by boosting prefrontal theta rhythm power. However, it remains unclear if the rhTMS protocol used to improve attentional control for neutral valence distractors can do the same for emotional distractors. Moreover, attentional control can be applied in different ways, either by amplifying relevant stimulus representation or by inhibiting distractor processing. In this study, we used rhTMS to increase theta power over the left dorsolateral prefrontal cortex in 19 healthy participants during a visual change-detection task. Auditory distractors with neutral and angry prosodies were heard during the task. Electroencephalography was used to measure theta power, contralateral delay activity, which indexes visual item maintenance, and the auditory P2, which indexes distractor processing. rhTMS boosted theta power and improved vWM accuracy compared to the sham condition. Theta boost was associated with lower emotional distractor processing, but not better maintenance. Our results indicate that the mechanism for attentional control of emotional auditory distractors in the vWM context is based on inhibition rather than amplification of relevant items.

## Introduction

Visual working memory (vWM) involves holding and manipulating a limited amount of visual information briefly (Williams *et al*. 2022, Ngiam 2024). Its performance has been linked to attentional control (Awh *et al*. 2006, Fukuda and Vogel 2009), which is the ability to prioritize goal-relevant information and suppress distractions. The neural basis of attentional control in vWM paradigms has been studied using techniques such as transcranial magnetic stimulation (TMS). Neurophysiological changes induced by TMS have been associated with changes in attention and vWM performance (Sauseng *et al*. 2009, Feredoes *et al*. 2011, Zanto *et al*. 2011, Emrich *et al*. 2017, Riddle *et al*. 2020). Yet, evidence for enhancing vWM through attentional control modulation has only been observed with targets and distractors of neutral emotional valence. Showing such improvements with emotional distractors would be particularly relevant since these are known to influence attention significantly (Yiend 2010, Carretie 2014, Zsidó 2024). In this study, we sought to enhance attentional control over emotional and neutral auditory distractors via TMS, combined with electroencephalography (EEG), to investigate the impact of this improvement on vWM performance.

As mentioned, the neural basis of WM has been previously studied with TMS (see Johnson *et al*. 2021). Stimulating the dorsolateral prefrontal cortex (DLPFC) has been shown to improve WM performance with neutral target items (Vanderhasselt 2014, Bagherzadeh *et al*. 2016, Brunoni and Hoy *et al*. 2016, Beynel *et al*. 2019). One study showed TMS-related improvements in both spatial n-back and digit span tasks, emphasizing the DLPFC’s general role in WM (Bagherzadeh *et al*. 2016). A meta-analysis indicated that the increase in n-back task accuracy after TMS stimulation is small but statistically significant (Brunoni and Vanderhasselt 2014). Another meta-analysis showed that stimulating both the right and left prefrontal cortex (PFC) with transcranial direct current stimulation (tDCS) can enhance WM (Wischnewski *et al*. 2021). These benefits from TMS and tDCS have been observed in experimental paradigms without distractors.

Other TMS studies demonstrate that the PFC allocates cognitive resources toward targets and away from distractors in vWM paradigms (Feredoes *et al*. 2011, Zanto *et al*. 2011, Lorenc *et al*. 2015, Riddle *et al*. 2020, Hallenbeck *et al*. 2025). The first cognitive control mechanism can be qualified as “target amplification” and the second as “distractor inhibition” (Noonan *et al*. 2016, Chang and Egeth 2019, Schneider *et al*. 2022). Feredoes *et al*. (2011) showed that perturbing the right DLPFC with TMS increased parietal activity related to visual target processing in a delayed-recognition task, but crucially only when distractors were present, suggesting that the PFC allocates resources to enhance target representation in the presence of irrelevant distractors. Similarly, Hallenbeck *et al*. (2025) found that stimulating the left PFC disrupted item prioritization in a memory-guided saccade task, as the performance for relevant and irrelevant items was similar after TMS. Zanto *et al*. (2011) showed that disrupting the inferior frontal junction with TMS decreased vWM accuracy and reduced the visual P1 amplitude evoked by target items, a marker of attentional resources. Riddle *et al*. (2020) demonstrated that TMS targeting the left middle frontal gyrus improved vWM by amplifying retro-cued item representations during a change-detection task. Overall, the PFC, including DLPFC, plays a key role in attentional control during vWM processing.

Previous TMS studies on vWM focused on targets and distractors with neutral emotional valence (e.g. Feredoes *et al*. 2011, Zanto *et al*. 2011). Some evidence suggests the DLPFC also processes emotional stimuli. For instance, stimulating the right DLPFC with low-frequency rTMS increases early magnetoencephalographic global field power evoked by fearful pictures (Zwanzger *et al*. 2014). The same parameters enhance the autonomic nervous system response to negative pictures (Berger *et al*. 2017). In a WM letter span task with visual distractors, modulating left DLPFC activity with tDCS influences emotional distractors’ impact on performance (Wolkenstein and Plewnia 2013, Wolkenstein *et al*. 2014). Performance improved with anodal tDCS (Wolkenstein and Plewnia 2013) and worsened with cathodal tDCS (Wolkenstein *et al*. 2014).

Non-invasive brain stimulation (NIBS) parameters are crucial in determining whether modulating DLPFC activity enhances or impairs vWM performance, both with tDCS and TMS. One key parameter for TMS is the stimulation frequency. Different frequencies can be used to entrain specific EEG rhythms using rhythmic TMS (rhTMS) (Bergmann *et al*. 2016). Enhancing specific EEG rhythms linked to cognitive function with rhTMS can lead to cognitive improvements (Luber and Lisanby 2014), such as increasing theta rhythm in WM paradigms (for review, see Albouy *et al*. 2018). For instance, rhTMS targeting theta rhythm in the DLPFC has been shown to improve the prioritization of target stimuli over distractors in a vWM change-detection task, resulting in better performance (Riddle *et al*. 2020). Conversely, performance on visual change detection decreased when rhTMS was set to entrain the alpha rhythm (Sauseng *et al*. 2009, Riddle *et al*. 2020). EEG studies have also linked prefrontal theta rhythm with change-detection task performance (Adam *et al*. 2015, 2018). All these findings have been observed with neutral distractors.

This study aimed to determine whether rhythmic TMS applied to the DLPFC during a vWM task could enhance performance when distractors possess an emotional negative valence. The left hemisphere was targeted to replicate findings from Riddle *et al*. (2020). We employed a change-detection paradigm to assess vWM as participants listened to irrelevant auditory distractors. Emotional negative distractors, such as those with fearful prosody, have been linked to reduced WM accuracy compared to neutral distractors with neutral prosody (Kattner and Ellermeier 2018). Behavioral outcomes are often ambiguous, as declines could stem from either increased focus on targets or improved distractor inhibition. To clarify this, EEG recordings were used to extract two event-related potentials (ERPs): the Contralateral Delay Activity (CDA), which reflects visual target processing—its amplitude correlating with the number of items held in vWM up to the individual’s capacity (Vogel and Machizawa 2004, Luria *et al*. 2016)—and the auditory P2, related to distractor saliency (Pell *et al*. 2015, Thiffault *et al*. 2024), which serves to monitor auditory distractor processing.

We hypothesized that rhythmic TMS aimed at increasing theta rhythm over the left DLPFC would reduce the vWM performance gap between neutral and emotional auditory distractors, compared to sham TMS. A second goal was to determine whether rhythmic TMS improved vWM accuracy through better target processing, as indicated by CDA amplitude, or by reducing distractor saliency, as indicated by auditory P2 amplitude.

## Materials and methods

### Participants

Nineteen participants (two men), aged 18–30 (*M* = 23.4, SD = 4.0), were recruited via social media. Exclusion criteria included being left-handed, using medication (except birth control), or having neurological or psychological disorders like Post-traumatic stress disorder (PTSD) or Attention-deficit/hyperactivity disorder (ADHD). Other exclusion criteria were related to TMS contraindications (Rossi *et al*. 2009). Two participants were excluded from ERP analyses: one due to line noise and one due to TMS artifacts. The experimental protocol was approved by the IRB at Université du Québec à Trois-Rivières.

### Design and general procedure

Each participant attended two sessions in the laboratory: one for the real TMS condition and the other for the sham condition. The order was counterbalanced across participants. Participants were blinded to the TMS condition.

The experiment proceeded as follows. The participants completed the consent form, followed by measurement of the motor threshold (MT), which was performed only in the first session due to its high test–retest reliability (Hermsen *et al*. 2016). After establishing MT, we fitted the EEG cap and the insert earphones. Participants then performed a change-detection task, comprising 700 trials (preceded by 12 practice trials).

**Figure 1:**
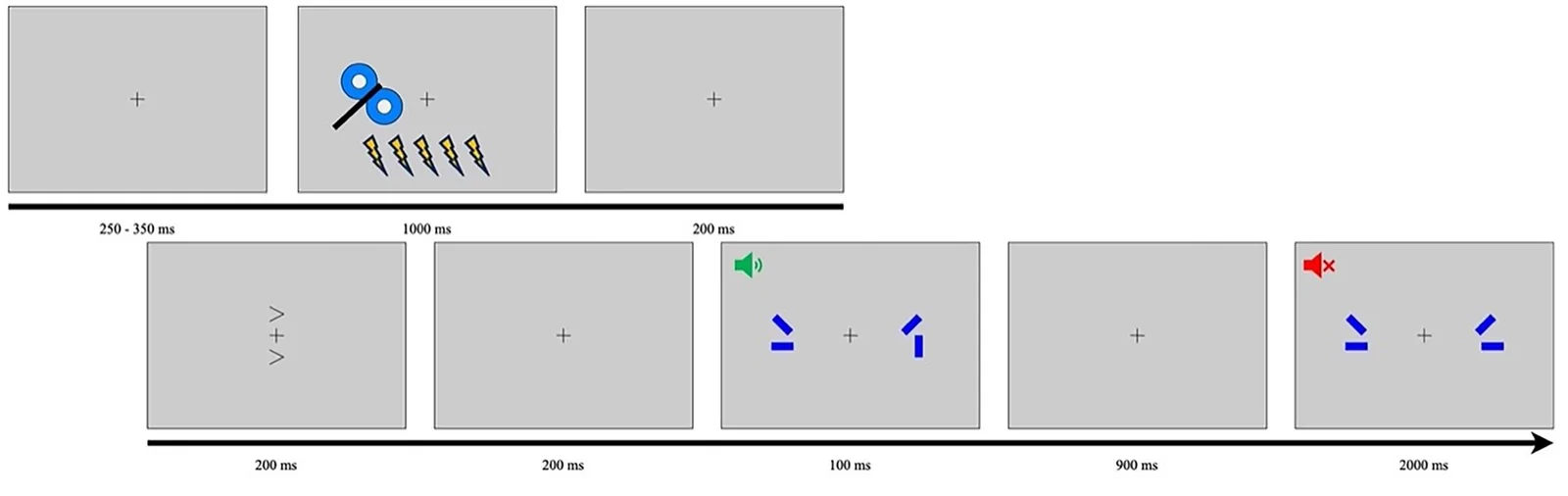
Rhythmic TMS burst and bilateral change-detection task trial. Before the task, a burst of five pulses was sent for 1 s (5 Hz). The task begins with arrows indicating the set of rectangles to memorize for 200 ms. After another 200 ms, the visual ensembles were presented for 100 ms. Auditory distractors started with the appearance of the rectangle sets. The target set was maintained in vWM for 900 ms. The recognition set was then presented. Auditory distractors stopped at the same moment. Participants had to detect if the orientation of one rectangle from the target set had changed or not.

Each trial in the change-detection task was preceded by a burst of five TMS pulses, each lasting 1000 ms, delivered at 5 Hz (see Fig. 1). A minimum inter-burst interval of 2000 ms was maintained between trials to reduce the risk of seizure from the TMS bursts. Participants could start the next trial by pressing the “spacebar” whenever they felt ready, following a minimum response time of 2000 ms. After pressing the spacebar, a jitter interval of 250–350 ms occurred before the 1000 ms TMS burst. An additional 200 ms buffer was added after the TMS burst to account for eye blinking following the last pulse (see Fig. 1). The entire task lasted ∼1.5 hours. Participants were informed of their progress after completing each block of 20 trials. Because they initiated each trial, participants could take a break whenever they desired.

### Change-detection task

A bilateral change-detection task, whole-display version, was used to measure vWM performance (Luck and Vogel 1997, Vogel and Machizawa 2004). The task began with the display of two arrows above and below a fixation cross for 200 ms. Arrows indicated which side was of interest: either left or right. Participants were instructed to focus on the indicated side without moving their eyes from the fixation cross. Next, two sets of rectangles appeared simultaneously to the left and right of the fixation cross for 100 ms. This marked the start of auditory distractor presentation. Sets could contain either two or four rectangles (corresponding to low and high working memory loads). Rectangles were oriented at 0°, 45°, 90°, or 135°. After the presentation, the sets disappeared, and a blank screen was shown for 900 ms. Upon reappearance, one rectangle from the set on the side of interest might be in a different orientation. Auditory distractors ceased when the rectangles reappeared. Participants were asked to determine whether there was an orientation change or not and responded by pressing “one” for a change or “two” for no change on the number pad with their right hand. The task was programmed using E-Prime 2.0 software (Psychology Software Tools, USA). The display was a 23-inch HP EliteDisplay E232 monitor. The viewing distance was 70 cm, with a resolution set to 1920 × 1080 at 60 Hz.

### Auditory distractors

We selected 20 pseudo-utterances in Hindi, from a pool of 40, for each of the two emotional distractor categories (angry or neutral) (Pell *et al*. 2009). We chose pseudo-utterances in Hindi to avoid evoking French or English words. Eleven members of our laboratory rated the arousal level of these distractors on a scale from one (“low arousal”) to five (“high arousal”). We also asked raters to identify the emotional category of the stimuli by choosing from four options. Neutral and angry distractors were correctly recognized, on average, at 89% and 66%, respectively, well above the chance level (25%). We selected the 20 pseudo-utterances with the lowest arousal ratings for neutral distractors (*M *= 1.06, SD = 0.04) and the 20 with the highest arousal ratings for angry distractors (*M *= 3.34, SD = 0.31). Participants heard distractors using Etymotic ER3C insert earphones (Etymotic Research, USA).

### Transcranial magnetic stimulation

#### Motor “hotspot” and threshold

MT was defined as the stimulator intensity evoking a motor evoked potential (MEP) of 50 µV of the right abductor pollicis brevis muscle when a single TMS pulse was sent over the left primary motor area “hotspot.” The motor hotspot is the area of the primary motor cortex that generates the highest MEP for a given intensity. To be confirmed as the MT, the stimulator intensity had to provoke an MEP of around 50 µV in 5 out of 10 attempts.

#### Pre-trial TMS burst

A 1-s TMS burst was sent 200 ms before the beginning of a change-detection trial. Stimulation intensity was set at 110% of MT. The TMS burst consisted of five pulses spaced by 200 ms (5 Hz). Minimal inter-train intervals were 3.85 s (see Fig. 1 for details regarding inter-trial/inter-train interval). The TMS coil was positioned over the F3 position of the 10-20 international system of EEG electrode placement. The stimulator used was the Magstim Rapid2 Plus1 (Magstim, UK) with the Magstim 2 Generation Air-Cooled Double 70 mm Coil. The sham condition consisted of tilting the coil 90° to the magnetic field plane.

### EEG recordings and analysis

#### Acquisition

EEG data were recorded using a TrueScan 64-channel amplifier (DEYMED Diagnostic, Czech Republic). Data were recorded at 1 kHz rather than the typical 5 kHz in TMS-EEG studies, as we were not interested in early TMS-evoked potentials. The ground electrode was installed at FPz, and the online reference electrode was on the left mastoid. Another electrode was installed on the right mastoid to create an average mastoid reference during offline analysis. Two electrodes, Fp2 and one installed 1 cm below the right eye, were used to create a bipolar vertical electrooculogram (VEOG) channel. Two other electrodes were placed 1 cm from the left and right outer canthi to form a bipolar horizontal electrooculogram (HEOG) channel.

#### TMS-related artifact removal and correction

We began by manually inspecting the data to identify and remove bad channels using BrainVision Analyzer 2 (version 2.3.0, Brain Products, Germany). Then, data were exported in MATLAB 2021b (MathWorks, USA) to utilize the TMS-EEG Signal Analyzer (TESA) EEGLAB plug-in and the TMS artifact correction protocol described by Rogasch *et al*. (2017). TMS pulse time points were found, and data were segmented from −1400 to 1750 ms relative to the last TMS pulse. Data around the TMS pulses (from −10 to 25 ms), containing TMS ringing artifacts and part of the TMS-evoked muscle activity, were removed and replaced with zeros. These segments were then demeaned before applying the independent component analysis (ICA). ICA was performed using the Fast ICA algorithm (Hyvärinen 1999) to eliminate remaining TMS-evoked muscle artifacts (25–50 ms) and to correct for long-lasting (over 500 ms) TMS decay artifacts.

After ICA correction, missing data around TMS pulses were interpolated using a cubic fit. Then, a median filter of order 100 was applied around TMS pulses (−20 to −40 ms) to remove remaining TMS-evoked muscle activity. The median filter order indicates the number of samples on each side of a data point used to calculate the median. A zero-phase Butterworth low-pass filter of order four (cutoff at 50 Hz) was used to eliminate high-frequency noise (such as power line interference and persistent muscle activity). A second ICA correction was performed to address eye blinks and eye movements. Channels removed at the start of preprocessing were reconstructed through interpolation.

#### Time-frequency-related preprocessing and analysis

A linear detrend was applied to eliminate low-frequency drift. Subsequently, data were imported into MNE-Python 1.7.0 for time-frequency analysis (Gramfort *et al*. 2013). Segments with peak-to-peak amplitude exceeding 120 μV were considered to contain residual TMS-related artifacts and were excluded. On average, 17% of segments were rejected. The participant with the fewest segments had 358 remaining segments. After removing bad segments, the data were re-referenced to the average.

A series of Morlet wavelets with frequencies from 2 to 40 Hz in 1 Hz steps was used to extract time-frequency activity from averaged segments, with each wavelet having five cycles. To extract time-frequency information with Morlet Wavelets without introducing ripple artifacts, segments were extended with mirror images (Cohen 2014). Power estimates were averaged across bands: theta (4–6 Hz), alpha (8–12 Hz), beta (12–30 Hz), and gamma (30–40 Hz). The two analysis windows were centered on rhythmic TMS stimulation: one from −100 to 900 ms relative to the first TMS pulse to verify entrainment during stimulation, and another from 100 to 900 ms relative to the last pulse to assess persistent entrainment after stimulation, covering three theta cycles.

#### ERP-related preprocessing and analysis

For ERP analyses, we reimported data in BrainVision Analyzer 2. We applied a different low-pass filter (half-power cut-off of 5 Hz) on channels that recorded electrooculogram (EOG) activity. After re-referencing the data to the averaged mastoids, we segmented data for 1050 ms starting 200 ms before the first rectangles set presentation. Segments were baseline corrected using the first 200 ms.

Segments containing a min-max difference over 80 µV in a 200 ms interval were identified as contaminated with movement artifacts. This criterion was applied on PO7 and PO8 for CDA extraction, Fz for P2 extraction, and other channels with the individual channel mode. Individual channel mode meant that contaminated segments were removed on those channels only, not on the others. The remaining vertical EOG activity (i.e. blink) following the ICA correction described in the previous section was detected as a min-max difference of 50 µV in a 200 ms interval on the right VEOG channel. Segments were also inspected for remaining horizontal EOG activity. For HEOG, the criterion was a min-max difference of 30 µV in a 150 ms interval. Following those inspection steps, an average of 6% of segments were rejected.

##### ERP components extraction

CDA was calculated as the difference between the average contralateral waveform and the ipsilateral lateral one. Then, a pooled activity of the left and right hemispheres was created for electrodes PO7 and PO8. The mean activity from 400 to 800 ms was used to calculate the CDA amplitude.

A peak detection algorithm was used to measure the auditory P2 component amplitude on average segments for the Fz channel (Pell *et al*. 2015). The algorithm was set to detect the peak positive amplitude between 150 and 300 ms. The peak activity was calculated as the mean of the 10 ms surrounding the highest values detected.

## Results

### Behavioral results

Accuracy scores for the change-detection task were entered in repeated-measures ANOVA with three within-subject factors: working memory load (two vs. four visual items), stimulation (active vs. sham), and distractor (neutral vs. angry). There was a significant main effect of working memory load, *F*(1, 18) = 107.77, *P <* .001, $\eta_{p}^{2}\;$= .86, such that the accuracy was higher when the working memory load was low (*M *= 0.79, SD = 0.06) than when it was high (*M *= 0.71, *SD *= 0.08). There was no main effect of stimulation, *F*(1, 18) = 2.11, *P=*.164, $\eta_{p}^{2}\;$= 0.10, nor of distractor type (*F* < 1).

**Figure 2:**
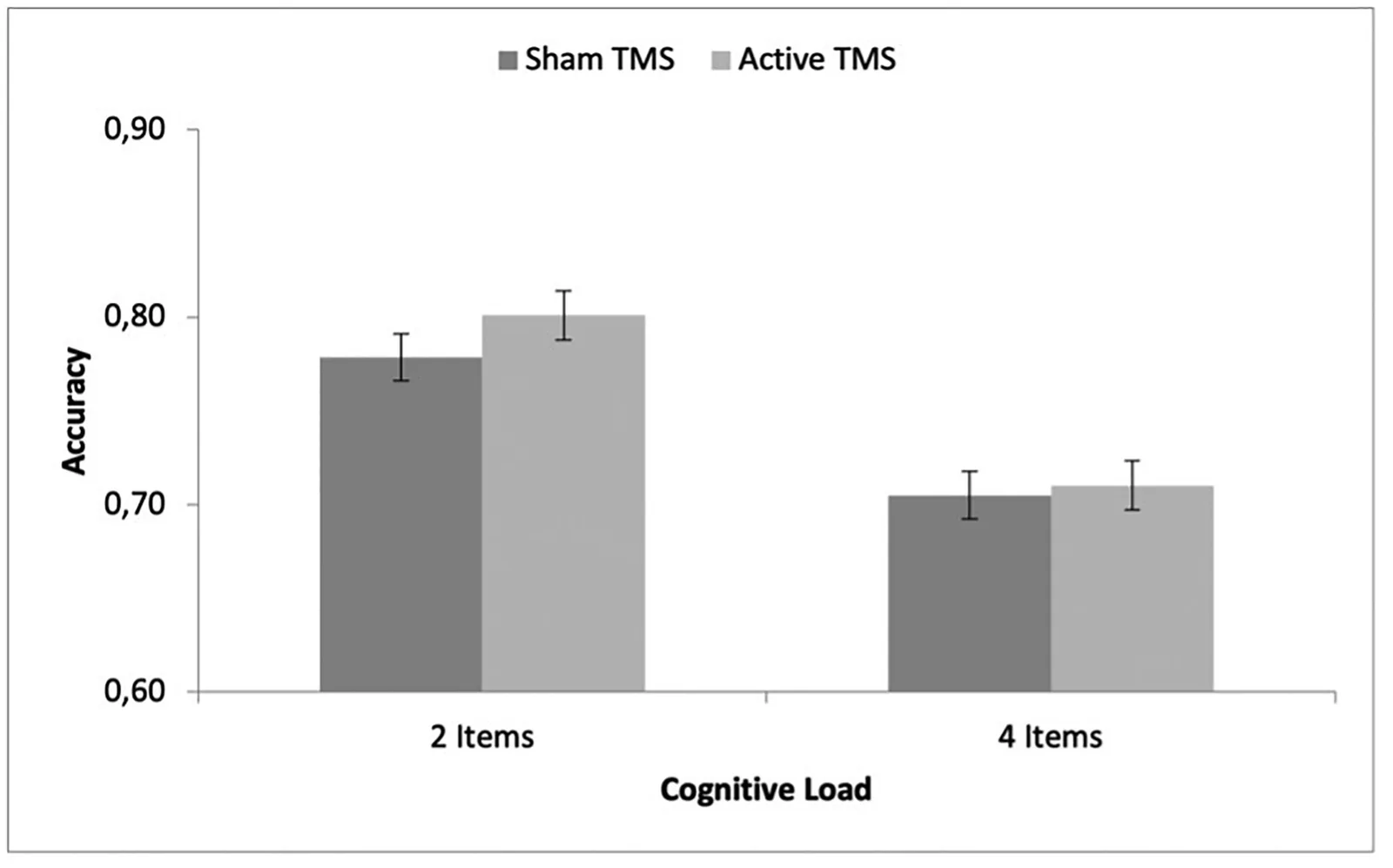
Change-detection accuracy scores as a function of stimulation and working memory load. Ninety-five percent confidence intervals are represented.

Working memory load and stimulation significantly interacted, *F*(1, 18) = 6.19, *P =* .023, $\eta_{p}^{2}\;$= .26. Post-hoc comparisons revealed that in the low working memory load condition, active stimulation led to higher accuracy (*M *= 0.80, SD = 0.07) compared to sham (*M *= 0.78, SD = 0.05), *t*(18) = 2.23, *d = *0.51, *P =* .039. This stimulation effect on accuracy was not seen in the high working memory load condition, *t*(18) = 0.52, *d = *0.12, *P =* .612 (see Fig. 2).

### Electroencephalography

#### Auditory P2

**Figure 3:**
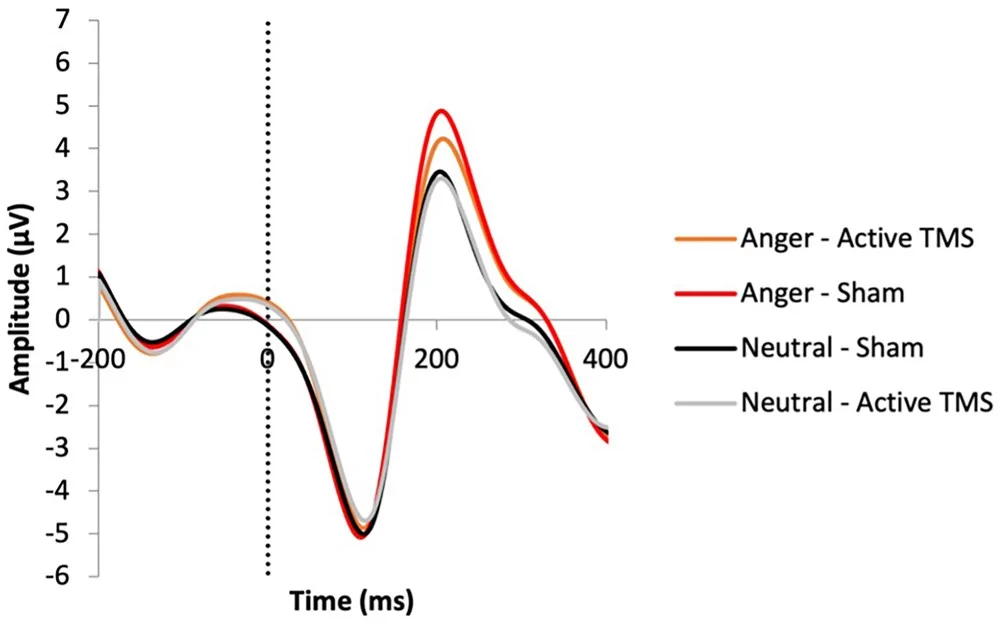
Auditory P2 components for all conditions of stimulation by distractor type.

A 2 × 2 × 2 ANOVA was used to examine the effect of distractors, stimulation, and working memory load on auditory P2 amplitudes. As expected, given that previous works showed no effect of visual perceptual and vWM load on early auditory components like the auditory N1 and Mismatch Negativity (Muller-Gass *et al*. 2006, Brockhoff *et al*. 2023a, 2023b), as well as the results our previous study (Thiffault *et al*. 2024), no effect of working memory load was observed (*F *< 1). Also expected because of previous studies (e.g. Pell *et al*. 2015), distracters with angry prosody produced auditory P2 with significatively greater amplitude (*M *= 6.78, SE = 0.46), compared to neutral prosody (*M *= 5.36, SE = 0.46) *F*(1, 16) = 21.53, *P* < .001, $\eta_{p\;}^{2}\;$= .57. P2 amplitude was lower in the active stimulation condition (*M *= 5.08, SE = 0.56) compared to sham (*M *= 7.07, SE = 0.56), (*F*(1,16) = 7.97, *P =* .012, $\eta_{p}^{2}\;$= .33). The interaction between distractor valence and stimulation was significant. The P2 amplitude produced by angry distractors was significantly lower in the active stimulation condition (*M *= 5.52, SE = 0.58) compared to sham *M *= 8.05, SE = 0.58), *t*(17) = 3.48, *d = *0.99*, P =* .016, (see Fig. 3).

#### Contralateral delay activity component

**Figure 4:**
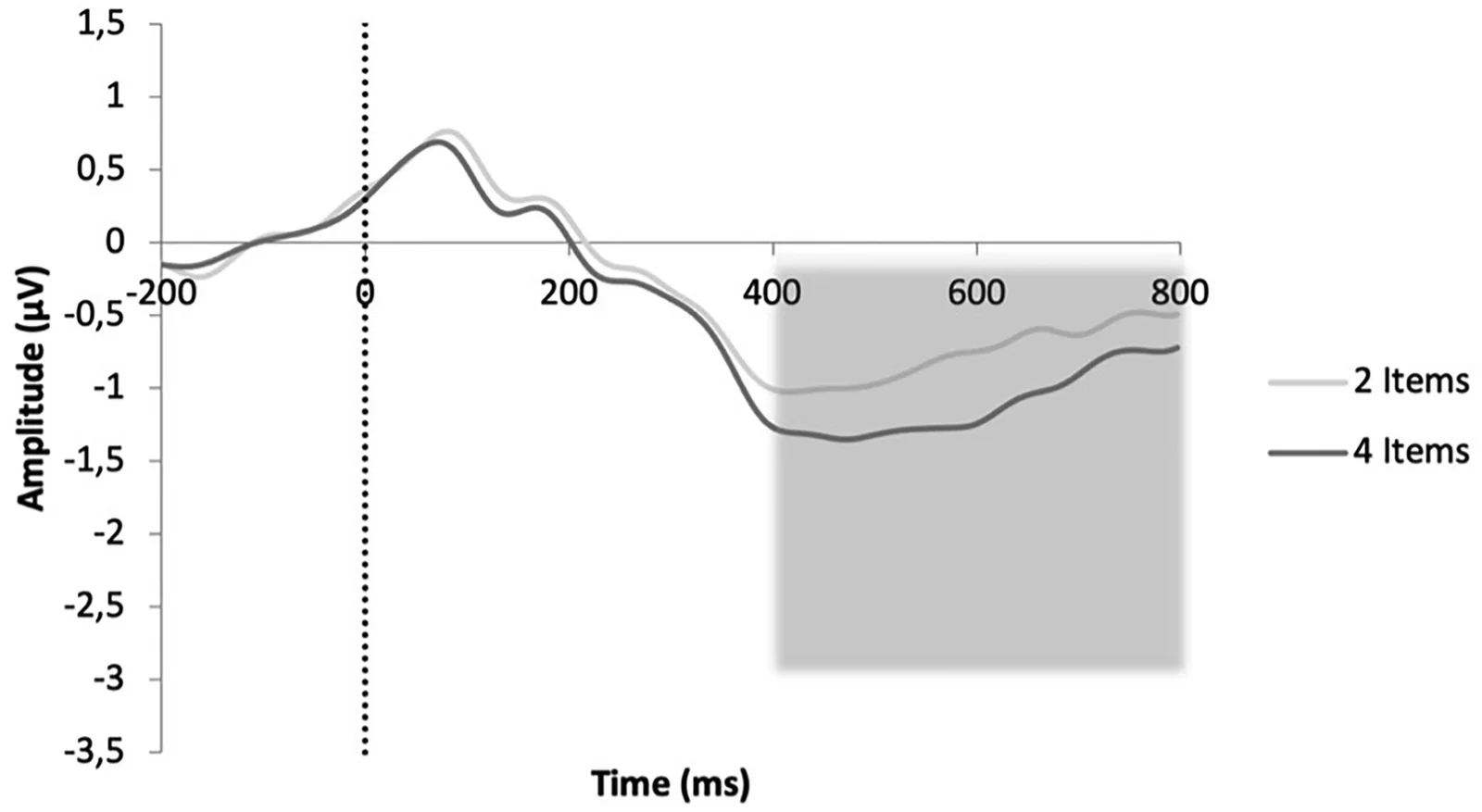
CDA components for low and high working memory load across all conditions of stimulation and distractor. CDA components were calculated from the mean activity of the shaded area.

We entered CDA amplitudes in a 2 × 2 × 2 repeated-measured ANOVA with the same variables as those included in the previous analysis. As expected (for review on the CDA, see Luria *et al*. 2016), higher working memory load trials generated greater negative amplitude CDA amplitudes (*M *=* −*1.11, SE = 0.30) compared to low working memory load trials (*M *=* −*0.76, SE = 0.22) *F*(1, 16) = 20.50, *p =* <.001, $\eta_{p}^{2}\;$= .56 (see Fig. 4). There was no effect of distractor valence (*F *< 1) nor stimulation (*F *< 1). No interactions were significant.

#### Time-frequency analyses

**Figure 5:**
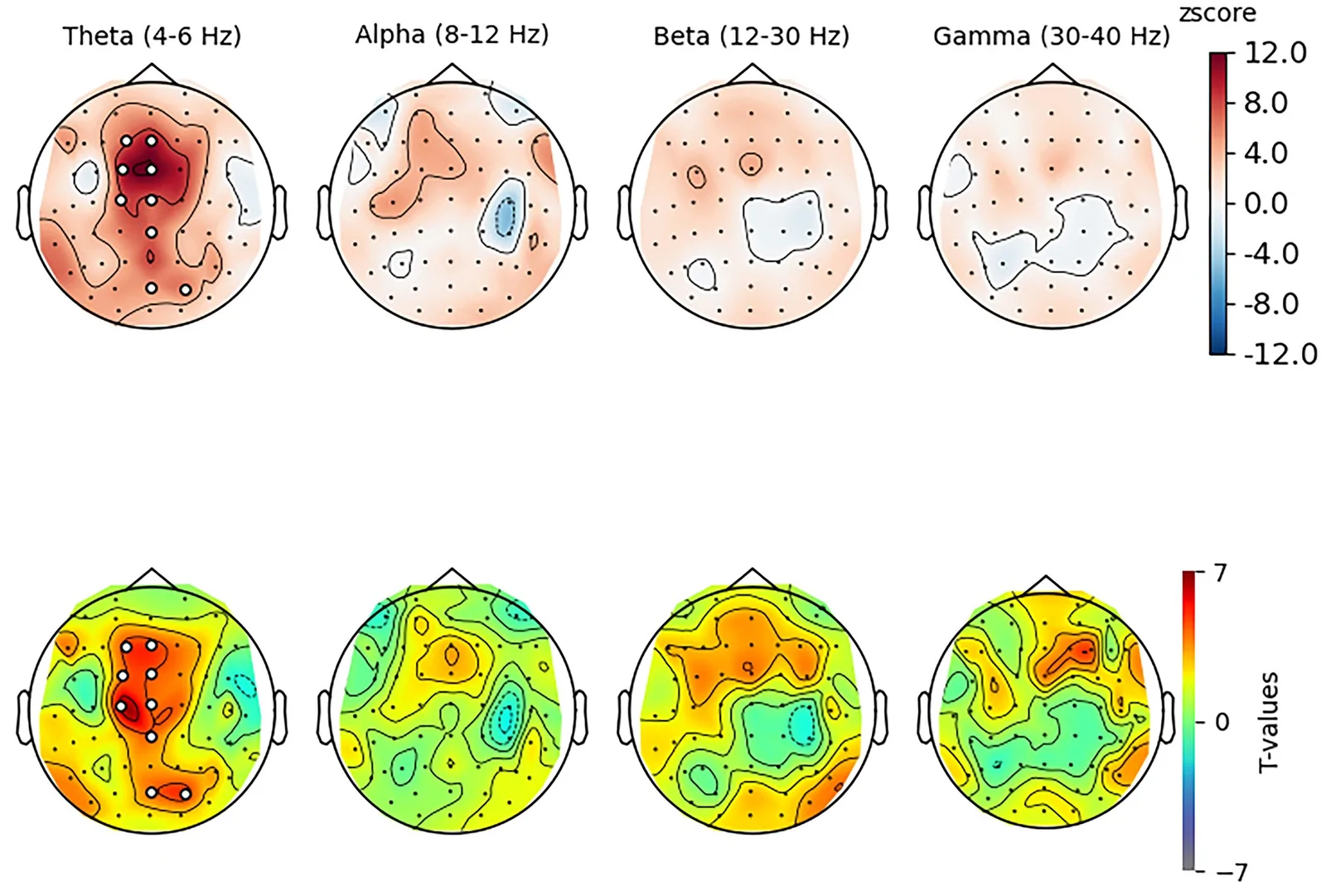
Topographies of power differences between sham and active stimulation with *t*-values clusters corrected. Channels significantly different (*P* < .05) are represented in white.

We extracted frequency activity evoked during and after pre-trial TMS bursts. Two average periods were chosen for statistical comparison: one from −100 to 900 ms relative to the first pulse of the TMS burst (stimulation period), and one from 100 to 700 ms following the last pulse of the TMS burst (post-stimulation period). Power values are represented as changes in z-score relative to a baseline period from −400 to −100 ms related to the first TMS pulse. We observed a significant increase in theta power in the stimulation condition relative to the sham condition during the TMS stimulation period. No significant power changes were observed in the other frequency bands. Using cluster-based statistics, we could determine that this change was mostly located in the left fronto-parietal region (Fig. 5).

**Figure 6:**
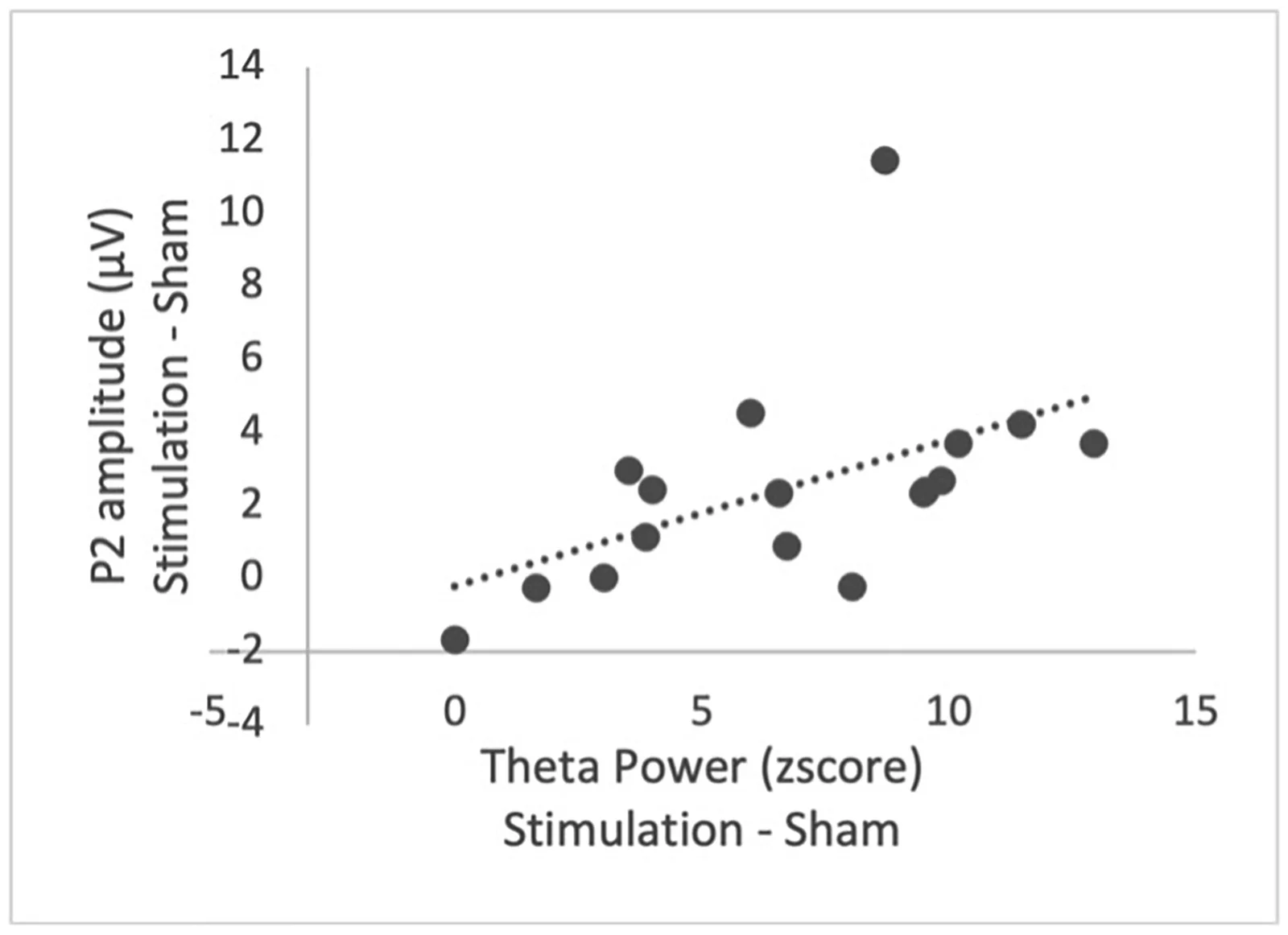
Relation between theta power and P2 amplitude difference scores. Difference scores compared the active and sham TMS conditions.

We examined whether the effect of stimulation on the salience of distractors could be related to the increased theta activity caused by the stimulation. Specifically, we calculated, for each participant, how much stimulation decreased P2 amplitude and correlated this with the increase in theta rhythm due to stimulation in the angry distractor conditions. The reduction in P2 amplitude after stimulation was significantly linked to higher theta power (*r *= 0.50, *P* = .018) (Fig. 6).

We tested if there was another association between the theta power increase, this time with vWM accuracy in the low working memory load. The power theta increase in that condition is moderately associated with vWM, but this was only marginally significant (*r *= 0.38, *P* = .064).

## Discussion

In this study, we aimed to determine whether stimulating the left DLPFC would improve vWM accuracy, especially in the presence of emotional auditory distractors. We found that rhTMS at 5 Hz improved accuracy in a low WM load condition (two targets) but not in a high load condition (four targets). Contrary to our hypothesis, improvement in vWM accuracy did not depend on whether emotional or neutral auditory distractors were presented. The rhythmic stimulation significantly increased the theta rhythm over the mid-central and fronto-parietal areas, and this increase in theta activity was linked to decreased attentional processing of emotional distractors.

We employed both ERPs and rhTMS to differentiate between two potential mechanisms underlying vWM enhancement. The CDA analysis did not support the idea that vWM improvements stem from better visual target maintenance, as rhTMS had no effect on CDA amplitude. An alternative explanation is that vWM gains may arise from reduced allocation of cognitive resources to auditory distractors, indexed by the auditory P2. This hypothesis received partial support: we found a significant reduction in the auditory P2 response to emotional distractors following rhTMS stimulation. Notably, this P2 decrease was linked to increased stimulation-induced theta power. Nonetheless, the changes in P2 amplitude did not correlate significantly with vWM accuracy, limiting our interpretation of the results.

The behavioral results of this study further support the effectiveness of online rhTMS in improving vWM, at least in the low working memory load conditions. This aligns with previous TMS vWM studies using change-detection tasks (Sauseng *et al*. 2009, Riddle *et al*. 2020). When both irrelevant and relevant stimuli were present in the vWM task, improvements were achieved either by inhibiting irrelevant stimuli processing (Sauseng *et al*. 2009, Riddle *et al*. 2020) or by enhancing relevant stimuli processing (Riddle *et al*. 2020). The method of improvement depended on TMS stimulation settings. For suppressing irrelevant stimuli, a stimulation within the alpha frequency range (10 Hz) was applied over the parietal region. Riddle *et al*. (2020) also tested boosting relevant stimuli representation by applying 5 Hz stimulation (theta range) to the left frontal cortex. The current investigation has been built upon the foundational findings of Riddle *et al*. (2020) by applying 5 Hz stimulation to the left DLPFC to enhance vWM by increasing the processing of relevant stimuli. The behavioral benefits of rhTMS appeared limited to the low vWM load condition. One possible explanation is that the high load itself can lessen the effect of distractors on performance, as suggested by the load theory of selective attention (Lavie *et al*. 2004, Macdonald and Lavie 2011). In other words, the high-load eliminated the need for better distractor control provided by the rhTMS.

The lateral prefrontal region, including the DLPFC, has been associated with target amplification rather than distractor inhibition in a vWM context, as shown by TMS (Feredoes *et al*. 2011, Zanto *et al*. 2011, Lorenc *et al*. 2015, Riddle *et al*. 2020). This differs from the attentional control mechanism we observed. Using auditory and visual ERPs, we found that our stimulation protocol decreased emotional distractor processing and did not enhance visual target maintenance. Indeed, rhTMS reduced the amplitude of the auditory P2 component, a component linked to prosody detection, elicited by the auditory distractor (Paulmann and Kotz 2008, Iredale *et al*. 2013, Pell *et al*. 2015, Wickens and Perry 2015). Our previous research showed that a decrease in the P2 amplitude during a similar task was associated with increased cognitive resources allocated to vWM maintenance (Thiffault *et al*. 2024). Furthermore, the decrease in P2 amplitude was correlated with prefrontal theta activity, an indicator of attentional control over distractors in WM (Sauseng and Liesefeld 2020). Given the small sample size, this correlation may have been spurious. Future research should aim to replicate the correlation between theta and distractor processing with a larger sample. Notwithstanding the small sample size, the fact that the change in P2 amplitude did not significantly correlate with the improvement in vWM accuracy, and that the correlation between theta increase and accuracy was only marginally significant, suggests that the theta-P2 pathway might be one of several mechanisms contributing to performance. This is in line with the dual-competition framework (Pessoa 2009), which indicates that emotional distractors not only bias perceptual processing, measured in this study using auditory P2, but also executive processes.

Other works using methods other than TMS have shown that the inhibition mechanism is a critical factor in vWM performance (reviewed by Lorenc *et al*. 2021). For example, the ERP index of active distractors suppression in a visual search task is associated with high vWM capacity (Gaspar *et al*. 2016). When measured with fMRI, pre-trial neural activity in the PFC has been associated with vWM performance and, more precisely, with distractor filtration (McNab and Klingberg 2008). The pre-trial is the period during which we applied stimulation, compared to the maintenance period, as done in the other vWM TMS studies mentioned above.

The cross-modal nature of our study might explain why we observed a different mechanism of cognitive control for distractors than in previous vWM-TMS studies, in which irrelevant and relevant stimuli were visual items or features. However, the previous vWM-TMS studies used fMRI, which has poorer temporal resolution than EEG, limiting comparison with our study. Keitel *et al*. (2013) used EEG steady-state responses, an early attention index, to study inhibition and facilitation in a cross-modal selective attention task. Visual targets weren’t amplified when shifting focus to visual stimuli alone, but auditory processing was inhibited. In contrast, cross-modal selective attention fMRI studies have shown that both inhibitory and facilitatory mechanisms are engaged when focusing on one sensory modality (Johnson and Zatorre 2005, Mayer *et al*. 2009), as well as DLPFC activation (Mayer *et al*. 2017).

One original aspect of our study was to present distractors with emotional valence, building on NIBS studies that used either neutral (Riddle *et al*. 2020) or emotional (Vanderhasselt *et al*. 2013) distractors. We found the same cognitive control substrate for both distractor types, consistent with neuroimaging studies (Banich *et al*. 2009). Our demonstration of a similar cognitive control substrate for emotional and neutral distractors would have been stronger if emotional distractors affected WM. However, despite selecting the most arousing emotional stimuli available, no impact of emotion on vWM was found. Emotional bias in cognitive tasks is hard to observe in healthy participants, as threat level may be perceived as low (Pessoa 2009). Future research could use aversive pairing to evoke stronger emotional responses and collect participants’ subjective ratings of the emotional valence and arousal ratings.

Due to limitations in our experimental design, it cannot be ruled out that neurophysiological mechanisms other than theta activity entrainment might contribute to improving vWM accuracy. Including an additional control condition, such as an arrhythmic stimulation burst, would be valuable for demonstrating that successive TMS-evoked activity, compared to rhTMS-induced entrainment, is insufficient to improve vWM, as previously shown (Riddle *et al*. 2020). An arrhythmic condition would also demonstrate that only rhTMS increases a specific EEG frequency band, as reported elsewhere (Thut *et al*. 2011, Albouy *et al*. 2017, Lin *et al*. 2021). Using a control frequency would show that the effect is specific to theta-band improvement (Bergmann and Hartwigsen 2021). An alternative neurophysiological mechanism is the increased theta band fronto-parietal connectivity following rhTMS, an effect lasting longer than the theta power enhancement (Albouy *et al*. 2017). This phenomenon has also been observed with offline theta-burst TMS on an n-back task (Hoy *et al*. 2016). Additionally, mechanisms not directly linked to rhythmic activity have been associated with vWM. A recent n-back task study revealed that single-pulse TMS applied to the left DLPFC increased WM accuracy by deactivating the dorsomedial default mode network (Webler *et al*. 2022).

To enable other researchers to reproduce this work with a better signal-to-noise ratio, it is recommended to use an EEG amplifier with a sampling rate higher than the 1 kHz used in the current work. A sampling rate of at least 3 kHz enables full capture of the TMS pulse artifact, facilitating artifact cleaning, especially TMS-induced muscle artifacts.

## Conclusion

Our findings demonstrate that rhTMS at 5 Hz improves the accuracy of vWM under certain conditions. Although other mechanisms cannot be completely ruled out based on the current results, the data are consistent with a role for an increase in theta-mediated auditory distractor inhibition, rather than target enhancement. Future research using different auditory and emotional distractors is needed to confirm these preliminary findings.

## Data Availability

The data underlying this article cannot be shared publicly because participants have not consented to do so.
